# Constipation With AMA and ANA Positivity as an Atypical Manifestation of Celiac Disease: A Case Report

**DOI:** 10.1002/ccr3.72535

**Published:** 2026-04-14

**Authors:** Sana Muhammad Hussain, Madiha Khan, Zahra Anas, Zarlish Khan, Amanullah Abbasi, Md Ariful Haque

**Affiliations:** ^1^ Internal Medicine Dr Ruth KM Pfau Civil Hospital Karachi Pakistan; ^2^ DOW University of Health Sciences Karachi Pakistan; ^3^ Voice of Doctors Research School Dhaka Bangladesh; ^4^ Department of Public Health Atish Dipankar University of Science and Technology Dhaka Bangladesh

**Keywords:** AMA antibodies, ANA antibodies, autoimmune disorder, celiac disease

**Key Clinical Message**

This case highlights that “celiac hepatitis” should be considered in patients with unexplained liver dysfunction, even when autoimmune markers are present. It underscores the importance of comprehensive clinicopathological correlation and raises awareness that timely recognition of CD can prevent misdiagnosis and allow reversal of liver abnormalities with appropriate treatment.

## 1 Introduction

Celiac disease (CD), also known as gluten‐sensitive enteropathy, is an autoimmune disorder caused by the ingestion of gluten in genetically susceptible individuals. While the small intestine is the primary site of involvement, resulting in symptoms such as diarrhea, flatulence, and weight loss due to malabsorption, CD is increasingly recognized as a systemic disease with potential involvement of the liver, thyroid, pancreas, connective tissue, bone, heart, skin, and nervous system [1].

Liver involvement in CD is well documented and includes a spectrum of disorders ranging from asymptomatic elevations in liver enzymes (celiac hepatitis [CH]) to associations with autoimmune liver diseases, such as autoimmune hepatitis (AIH) and primary biliary cholangitis (PBC) [2].

AIH is a chronic progressive autoimmune liver disease characterized by immune‐mediated hepatocellular inflammation, which may lead to cirrhosis and liver failure. It primarily affects women and has an estimated incidence of 0.85–1.68 per 100,000 person‐years [3] In another study conducted by Hahn et al., the incidence and prevalence of AIH were found to be 1.68 persons per 100,000 person‐years [4]. AIH is differentiated into Type 1 AIH and is linked with antinuclear antibodies (ANA) and/or smooth muscle antibodies (SMA), and Type 2 AIH is associated with anti‐liver/kidney microsome Type 1 (anti‐LKM‐1) and/or anti‐liver cytosol Type 1 antibodies [5]. While these autoantibodies aid in diagnosis, they lack absolute disease specificity, as they may be present in other conditions or even in healthy individuals.

PBC, another autoimmune liver disorder, is serologically defined by the presence of anti‐mitochondrial antibodies (AMA), which, when accompanied by a cholestatic biochemical profile, typically confirm the diagnosis without the need for liver biopsy. In patients with CD, the most frequently observed hepatic abnormality is CH, often presenting as a mild, reversible elevation in aminotransferases in response to a gluten‐free diet [6].

Here, we present a rare case of a 40‐year‐old woman with confirmed CD and associated CH who demonstrated concurrent positivity for AMA and ANA serological markers suggestive of PBC and AIH, respectively, yet lacked the clinical and histopathological features necessary to diagnose either condition. This case highlights the complexity of serological overlap in autoimmune conditions and underscores the importance of thorough clinical correlation in CD patients.

## 2 Case Presentation

In 2021, a 40‐year‐old female weighing 55 kg and 5 ft 5 in. tall, with no previous comorbidities, presented to the outpatient department with complaints of constipation for 5 years, heavy menstrual bleeding for 2 years, multiple joint pain for 1 year, and weight loss for 1 year. She reported passing one hard stool within 3–4 days, which was not mixed with mucus or blood, and there was no history of fresh bleeding per rectum. Defecation was sometimes associated with lower abdominal pain, mild to moderate in intensity, crampy in nature, and non‐radiating. There are no known aggravating factors of pain.

The patient reported no history of heat or cold intolerance, tenesmus, or change in dietary habits. Over the past 2 years, she has experienced heavy menstrual bleeding, characterized by passing clots, lasting more than 3–4 days, and requiring pad changes three to four times daily, which was an increase from her usual cycle. The bleeding was accompanied by dysmenorrhea, but the patient denied pelvic pressure, intermenstrual bleeding, or recent contraceptive use.

**FIGURE 1:**
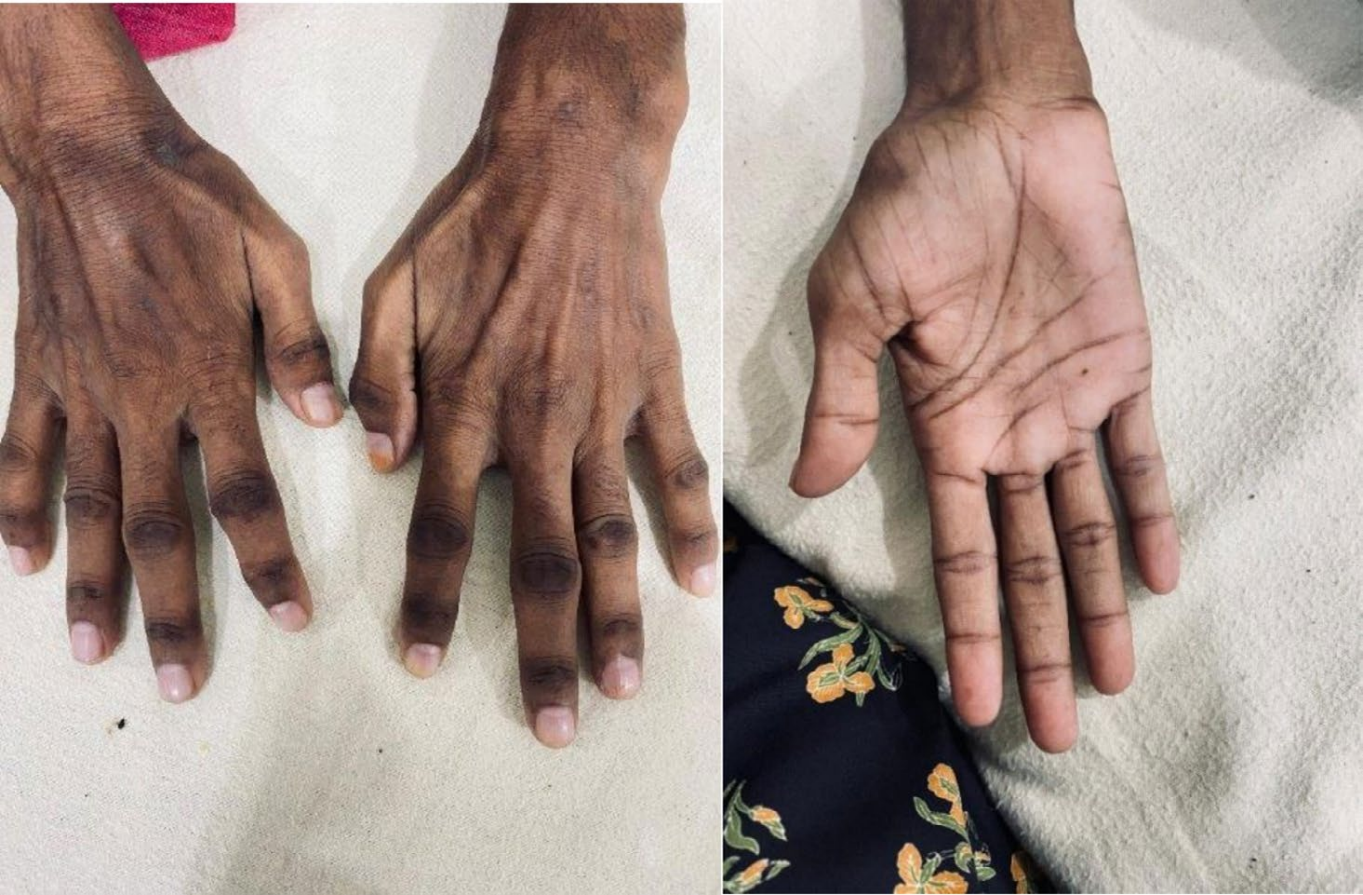
Darkening of knuckles, joints, and palmar creases.

She also described symmetrical joint pain in both the small and large joints, unrelated to movement or rest, which limited her daily activities. No morning stiffness, swelling, rash, Raynaud's phenomenon, photophobia, or photosensitivity was observed. Although rheumatoid arthritis was ruled out by negative anti‐CCP antibodies and rheumatoid factor, moreover, no radiological evidence was found in the hand x‐ray in Figure 3. However, she reported oral ulcers and excessive hair loss over the past year, along with occasional skin itching. She denied having dry eyes, dry mouth, dysphagia, skin tightening, or back pain. In addition, she noted unintentional weight loss, reduced appetite, and an undocumented low‐grade fever with night sweats over the past year (Figure 1).

**FIGURE 2:**
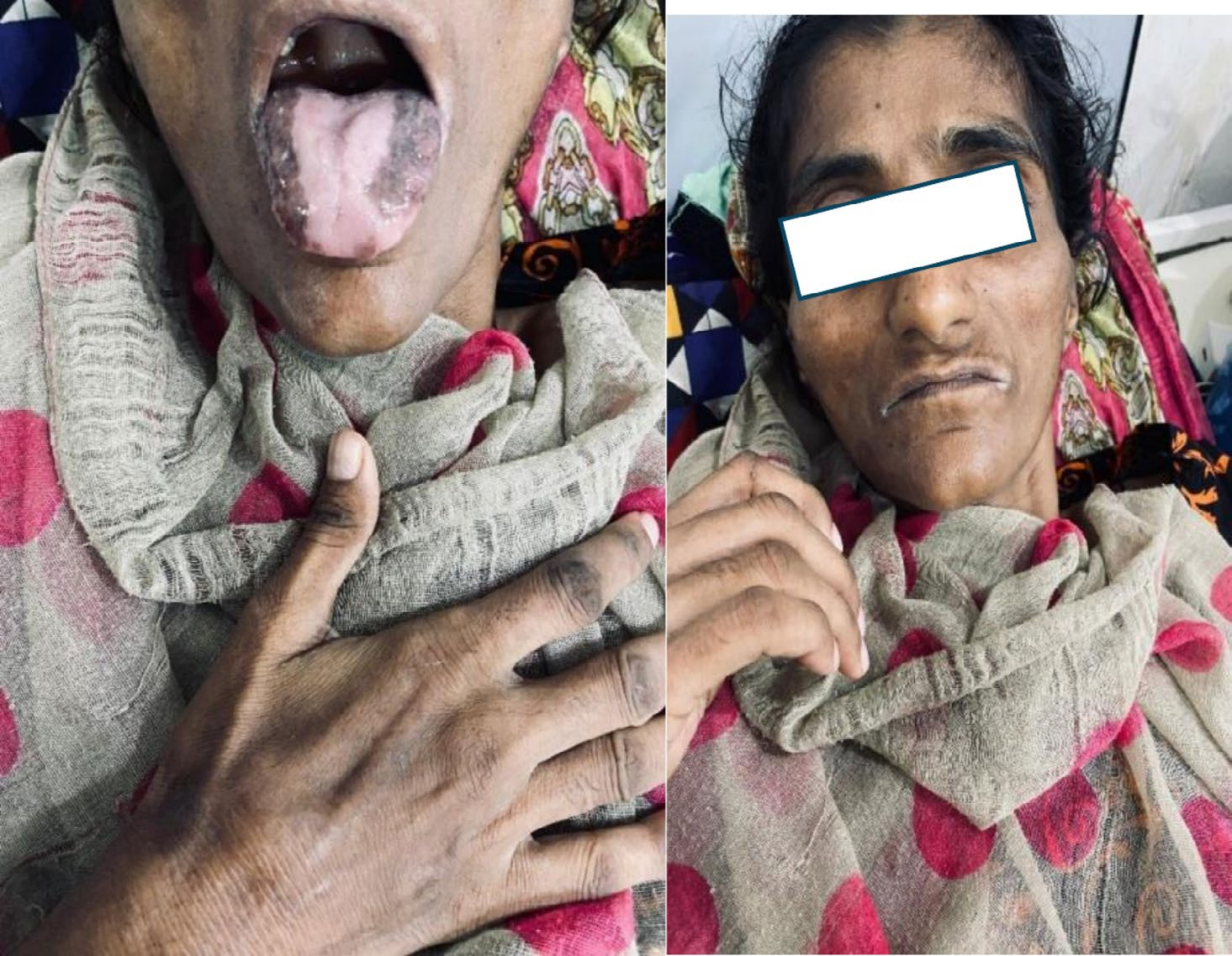
Hyperpigmentation on the lateral borders of the tongue.

**FIGURE 3:**
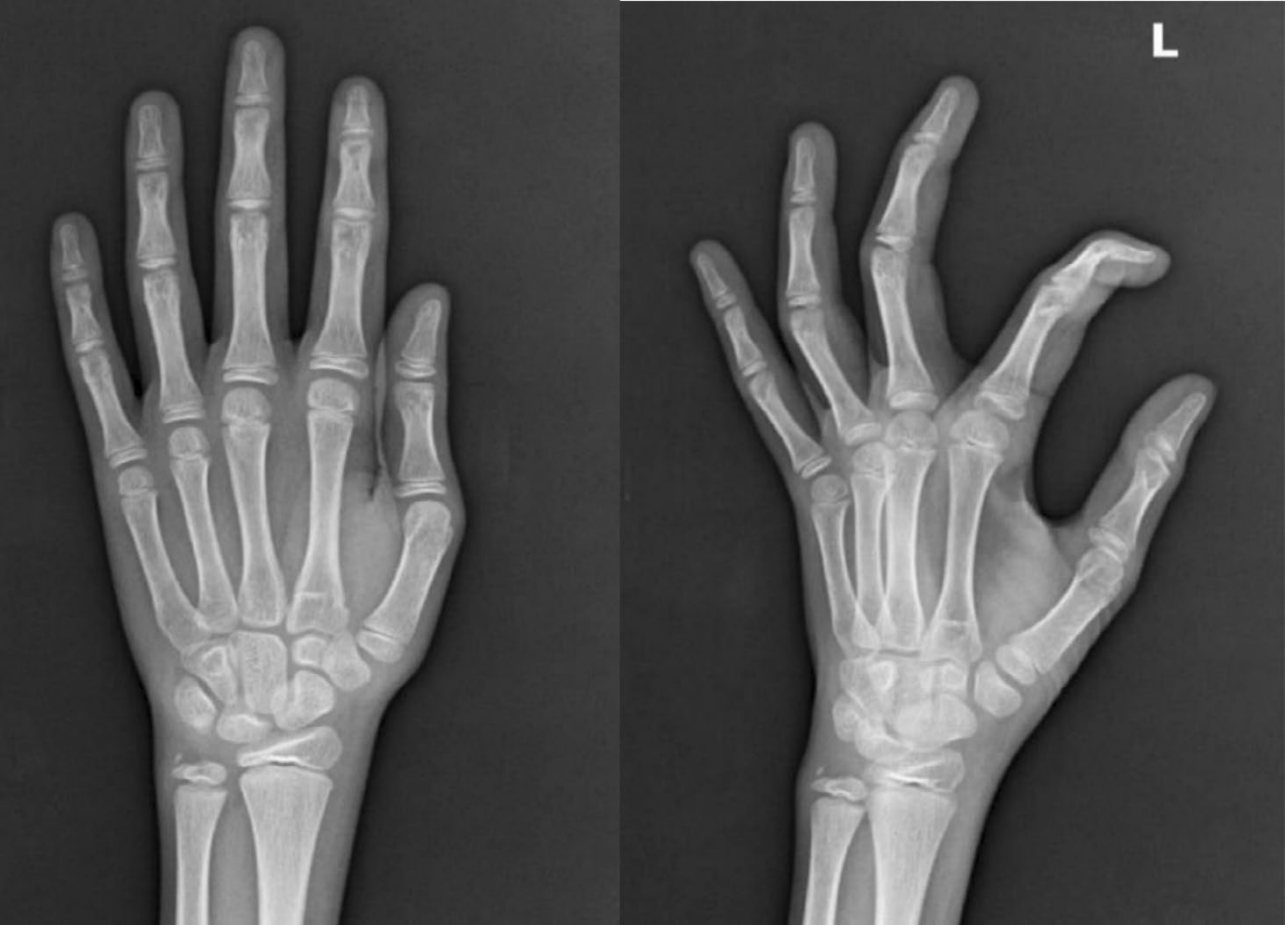
Normal x‐ray of hands (left and right) with no radiological evidence of rheumatoid arthritis/deformity.

Upon examination, an adult female with short stature and a lean, thin build, with buccal and temporal wasting, along with darkening of the skin and palmar creases, was observed (Figures 2 and 3). Excoriation marks were visible on her arms and legs. Her vital signs at the time of admission were a blood pressure of 100/70 mmHg without any postural drop, a pulse of 84 beats/min with regular rhythm, a respiratory rate of 16 breaths/min, and a temperature of 98.7°F (37°C).

## 3 Methods

Upon admission, laboratory investigations revealed pancytopenia with a hemoglobin of 8.6 g/dL, an MCV of 70.7 fL, and a platelet count of 130. Liver function tests revealed elevated enzyme levels with total bilirubin of 1.6 mg/L, AST 80 U/L, ALT 60 U/L, alkaline phosphatase 250 U/L, and GGT 160 U/L, showing a cholestatic pattern. The patient had hypoalbuminemia (2.8 g/dL) with hyperglobulinemia (4.4 g/dL), resulting in a reduced A/G ratio of 0.63. Further testing showed iron deficiency (20 μg/dL), a reactive hepatitis B core antibody with negative PCR results, and normal thyroid function and cortisol levels (Tables 1 and 2). Initial imaging studies included an abdominal ultrasound showing altered hepatic echotexture and a transvaginal ultrasound revealing an intramural fibroid; chest radiography and an abdominal CT scan were unremarkable.

**TABLE 1:** Various lab parameters on admission.

Hematology	Patient's labs	Normal range	Units
Hb	8.6	11.5–15.5	g/dL
MCV	70.7	80.0–100.0	fL
HCT	27.8%		
TLC	6.5	4.0–10.0	×10^9^/L
Lymphocytes	62	20.0–40.0	%
Neutrophils	28	40.0–80.0	%
Platelets	130	150.0–450.0	×10^9^/L
Iron	20	37–145	mcg/dL
TIBC	326	240–450	mcg/dL
Transferrin saturation	6.135%	20–50	%
B12	180	200–1000	ng/L
CRP	4.5	0 < 5	mg/L
ESR	60	0–20	mm/h
Prothrombin time	12.5	11–13	
aPTT	22.5	26–36	
INR	1.28		
Peripheral film	Anisocytosis, hypochromic, microcytosis, hypersegmented neutrophils		
Urine D/R	pH 6, specific gravity = 1.01, protein trace, normal quantity of urinobilogen, trace of blood, red cells: 8–10/hpf and pus cells 1–2/hpf		
Basic metabolic profile			
Component		Normal range	Units
BUN	6	7–20	mg/dL
Cr	0.3	0.7–1.3	mg/dL
Sodium	136	134–144	mEq/L
Potassium	3.8	3.5–5.2	mEq/L
Chloride	103	96–105	mEq/L
Calcium	7.2	8.7–10.2	mg/dL
Magnesium	2	1.7–2.2	mg/dL
Phosphate	3.5	2.5–4.5	mg/dL
FBS	95		mg/dL
HBA1C	5.9%		
Uric acid	3.8	3.5–7.2	g/dL
Total protein	7.2	6.4–8.2	g/dL
Albumin	2.8	3.4–5.0	g/dL
Globulin	4.4	1.9–2.8	g/dL
Albumin/globulin ratio	0.63	0.8–2.5	
TSH	1.4	0.4–4.2	IU/mL
T3	114.98	70–200	ng/dL
T4	9.48	4.5–11.5	mcg/dL
Serum cortisol	16.80	Morning 4.82–19.5	mcg/dL
Liver function tests			
Component		Normal range	Units
ALT	60*	7–55	U/L
AST	80*	5–40	U/L
ALP	250*	40–129	U/L
GGT	160*	< 35	U/L
Total bilirubin	1.6	0.1–1.2	mg/dL
Direct bilirubin	1.0		
Indirect bilirubin	0.6		

*Note:* *Indicates abnormality in lab values.

**TABLE 2:** Relevant autoimmune work‐up during hospital admittance.

Component	Result	Normal range	Units
Serum anti‐transglutaminase IgA	Positive (14.80)	Positive > 3.5	U/mL
ANA	1:160	Positive titre ≥ 1:160	
Anti‐CCP antibodies	< 20	Positive > 20	IU/mL
Rheumatoid factor	Negative	14–20	IU/mL
Serum anti‐DsDNA	6.42	Positive > 25	IU/mL
AMA‐M2 IgG	17 (positive)	Positive > 10	IU/mL
U1‐RNP‐Ab	Negative	Positive > 2.4	U/mL
SS‐A/RO‐AB	Negative	Positive > 12.5	U/mL
SS‐B/LA‐AB	Negative	Positive > 7	U/mL
Anti‐SM‐AB	Negative	Positive > 25	U/mL
Serum anticardiolipin IgG	10.55	< 15	U/mL
Serum anticardiolipin IgM	0.10	< 12.5	U/mL
Beta 2 glycoprotein 1 IgG and IgM	> 0.21/0.25	Positive > 1.0	U/mL

The diagnostic procedures included colonoscopy with normal mucosal findings up to the terminal ileum, although biopsies demonstrated mild nonspecific colitis. Esophagogastroduodenoscopy revealed scalloping of the duodenal folds, and histological examination of duodenal biopsies showed Marsh 3a lesions, characterized by villous blunting and increased intraepithelial lymphocytes. Serological testing confirmed CD with immunoglobulin A (IgA) tissue transglutaminase antibodies. Autoimmune evaluation yielded a positive ANA with a fine‐speckled pattern and positive AMA, whereas dsDNA antibodies, ENA profile, and antiphospholipid antibodies were negative (Table 3). Hepatic elastography indicated F2 fibrosis (Metavir score 9.0 kPa), with a liver biopsy demonstrating bland cholestasis and mild portal inflammation classified as Grade 1, Stage 0.

**TABLE 3:** Viral markers workup.

Viral markers	Reactivity
Hep B core	Reactive
HBsAg	Nonreactive
Hep B PCR	Negative
HBeAb	Nonreactive
Anti‐HCV	Nonreactive
Anti‐HDV	Nonreactive
HIV	Nonreactive

Abbreviations: HBeAb, hepatitis B envelope antibody; HBsAg, hepatitis B surface antigen.

The patient was diagnosed with AMA‐ and ANA‐positive CD with CH, presenting with atypical features after an extensive workup to rule out autoimmune illness, including liver biopsy; apart from ANA and AMA, all were negative, and the biopsy did not show features of any autoimmune disease. Management consisted of strict gluten‐free dietary modification and pharmacotherapy, including 25 mg hydroxyzine for pruritus, witha naproxen 550 mg and paracetamol/orphenadrine 650/50 mg combination as needed for pain control. Nutritional supplementation addressed malabsorption‐related deficiencies, including high‐dose chewable vitamin A (50,000 IU daily), vitamin E (400 mg daily), comprehensive multivitamins, and combined calcium with vitamin D3 and K2. This therapeutic regimen was implemented to address both the autoimmune and malabsorptive components of her condition while managing symptomatic complaints.

## 4 Discussion

CD is an autoimmune gastroenteropathy caused by gluten sensitivity, which leads to malabsorption resulting from villous atrophy. The main pathogenic factors are antibodies specific to gluten, which lead to intestinal and extraintestinal symptoms. Moreover, it is associated with other autoimmune conditions, such as thyroiditis, Type 1 diabetes, and rheumatoid arthritis. However, along with other organs involved in CD, the liver can develop AIH, PBC, primary biliary cirrhosis, or CH in approximately 2%–20% of children and the adult population [3].

According to a single‐center study, liver manifestations in CD occurred in about 75% of the population, with AIH in 11% and overlap of AIH/PBC and PBC alone in 3% of the cases [7]. Moreover, the typical clinical symptoms of CD include diarrhea, bloating, flatulence, and abdominal pain and can also present with constipation. According to a single‐center study, approximately 4.2% of children presented with constipation in celiac, with a higher prevalence compared to the group without constipation [3, 8]. Another recent cross‐sectional study by John et al. demonstrates that 45% of patients with CD who underwent Anorectal manometry (ARM) had constipation, concluding that it can be due to a compensatory mechanism of chronic diarrhea [9].

AIH is an inflammatory condition involving antibodies that can develop different forms of presentation varying across ethnicities and age groups, such as jaundice, cirrhosis, or liver failure with rapid recurrence of AIH after transplantation. The pathophysiology of AIH is attributed to antibodies, including antinuclear–nuclear antibodies, anti‐SMA, anti‐liver‐kidney microsomal antibody, or LKM antibody, depending on the type of AIH, although anti‐mitochondrial antibody (AMA‐M2) is predominant and highly diagnostic for PBC [10]. When PBC is compared to AIH, there is marked inflammation and hepatocyte necrosis, and no ductular reaction or florid duct lesion with lymphocytosis, unlike in PBC [7, 10].

CH is one of the extraintestinal manifestations of CD with mild liver steatosis and elevated liver enzymes; on biopsy, there is a mild to moderate portal or lobular inflammation with mononuclear infiltrate and steatosis, which can be termed cryptogenic hepatitis because there is no apparent cause of CH‐like viruses, AIH, PBC, PSC, and so forth. The only antibody found responsible for this is the anti‐TTG antibody, which rarely leads to cirrhosis [10]. In addition, CH is defined by hypertransaminasemia and/or histological changes in the absence of other causes, with resolution following a gluten‐free diet. In contrast, coexisting autoimmune hepatobiliary disorders typically do not respond to dietary therapy alone [11]. In this context, our case is notable for its atypical hepatic presentation with overlapping autoimmune serology yet features consistent with celiac‐related liver involvement and resolution of CH on GFD, a pattern rarely emphasized in the literature.

However, mild hepatic steatosis in CD has been associated with metabolic dysfunction‐associated steatotic liver disease (MASLD), previously known as nonalcoholic fatty liver disease (NAFLD), specifically when the patient also has cardiometabolic factors. Hence, it is highly important to screen for liver disease or injury with cryptogenic elevated transaminases in CD to rule out any metabolic syndrome or other etiologies present. In addition, a gluten‐free diet can help alleviate hepatic symptoms and enzyme levels in most cases, whereas in some contradicting data, it can lead to MASLD or steatohepatitis. This is because gluten‐free alternatives have a high glycemic index and lead to hyperlipidemia and triglyceridemia [12].

Our report describes an unusual case of a 40‐year‐old female with atypical CD presenting with constipation and CH, along with detectable AMA and ANA antibodies, yet lacking clinical features of AIH or primary biliary cirrhosis. According to the International Autoimmune Hepatitis Group (IAIHG), our case did not meet the criteria for AIH completely, with a score of < 4, whereas a score of ≥ 7 is required for a definitive diagnosis of AIH [13]. It does not fit the diagnostic criteria for PBC due to the absence of hepatocyte necrosis, ductular reaction, or florid duct lesion with lymphocytosis on biopsy [7]. However, biopsy results indicated CH. Only the peculiar presence of AMA‐M2 and ANA in diagnosed CD stands out with liver biopsy only, indicating bland steatosis consistent with CH.

Comparative analysis of the literature shows the hepatic manifestation of CD ranges in severity from mild reversible CH to severe hepatitis that requires immunosuppression. Moreover, it denotes that autoimmune serology in celiac‐associated liver dysfunction can range from a complete seronegative profile to the isolated presence of autoantibody without histopathological features of either AIH, PBC, or CH.

Kaptan et al. reported a case of CD and AIH that led to fulminant hepatitis. In addition, there was five times higher hypertransaminasemia and a liver biopsy that confirmed seronegative AIH without the presence of AMA and ANA antibodies and the development of fulminant hepatic failure. They managed it with prednisone and a gluten‐free diet, which was only responsible for alleviating hepatic symptoms in our patient [14]. With the CD‐AIH combination, patients showed a higher chance of remission and early withdrawal from antipsychotics as well as less severe liver symptoms [2].

CH‐led cirrhosis is rare, and secondary portal hypertension is unusual. Sawlani et al. addressed the case of a 35‐year‐old male with idiopathic portal hypertension secondary to CH and no association with AIH or PBC. In comparison, our patient did not develop any signs of portal hypertension, such as ascites or varices, and an altered echotexture of the liver with smooth margins, along with a patent portal vein on CT scan, although our case had a positive test for hepatitis B core antibody that could contribute toward a higher METAVIR score [15]. Similarly, Sood et al. also reported a young female with CD who developed idiopathic portal hypertension without cirrhotic changes in the liver. Contrary to the other case reports discussed above, there were no antibodies such as anti‐SLA, AMA, or ANA, or any histopathology indicating AIH or PBC [16].

However, the two cases reported by Demir et al. showed the most similarity to our case. The case report presented two female patients belonging to a younger age group (25–35) diagnosed with CD and with positive serology of anti‐SLA and AMA‐M2 antibodies, respectively. The histopathology in the first case was consistent with serology‐positive AIH, although no ductal injury was noted, whereas the second case showed liver‐injury‐associated CD. Both cases had elevated transaminase and ALP levels. ALP levels could also be increased due to decreased bone mineral density indicated by their DEXA scan results, although in our case, the ALP level was solely raised due to mild hepatic inflammation. GFD, prednisolone, and azathioprine were used to treat AIH and CD, and patients went into remission within 6–12 months [5]. The case reports are summarized and compared in Tables 4 and 5.

**TABLE 4:** Comparison of case reports: AMA positive celiac disease with liver involvement.

Case report author	Year published	Age	Gender	Liver involvement	Serology/antibodies	Histopathology	Final diagnosis	Treatment
Kaptan et al. [14]	2021	8	Female	Fulminant hepatitis post‐AIH	Seronegative AIH; AMA and ANA negative	Liver biopsy positive for AIH	Celiac disease + AIH → fulminant hepatic failure	Prednisone + gluten‐free diet
Sawlani et al. [15]	2024	35	Male	Idiopathic portal hypertension due to celiac hepatitis	No AIH or PBC; no antibody association	No cirrhosis	Celiac hepatitis with secondary portal hypertension	Gluten‐free diet
Sood et al. [16]	2024	22	Female	Idiopathic portal hypertension without cirrhosis	ANA, AMA, Anti‐SLA negative	No histopathology of AIH or PBC	Celiac disease with idiopathic portal hypertension	Gluten‐free diet
Demir et al. (Case 1) [5]	2020	25–35	Female	AIH with liver injury	Anti‐SLA positive	AIH‐like histopathology, no ductal injury	Celiac disease + AIH	GFD + prednisolone + azathioprine
Demir et al. (Case 2)	2020	25–35	Female	Celiac‐related liver injury	AMA‐M2 positive	Celiac‐associated liver injury	Celiac disease with autoimmune features	GFD + prednisolone + azathioprine

**TABLE 5:** Comparison of AIH, PBC, and celiac hepatitis parameters.

Parameters	AIH	PBC	Celiac hepatitis (CH)
Clinical features	Jaundice, hepatitis, cirrhosis	Pruritus, fatigue, cholestasis	Often mild or asymptomatic; linked to celiac disease
Biochemistry	↑ ALT, AST (hepatocellular)	↑ ALP, GGT (cholestatic)	Mild ↑ ALT/AST (±mixed pattern)
Serology	ANA, ASMA, anti‐LKM	AMA (AMA‐M2)	Anti‐tTG
Histology	Interface hepatitis	Bile duct destruction	Mild inflammation ± steatosis
Treatment response	Immunosuppressants	UDCA	Gluten‐free diet → resolution
Key point	Autoimmune hepatitis	Autoimmune cholestasis	Reversible liver injury with GFD

## 5 Conclusion

CD can lead to classic gastrointestinal symptoms, but there can be a rare extraintestinal manifestation involving the liver. Hepatic manifestations can be diverse in celiac patients, including CH, AIH, and PBC, as illustrated by the unique case of a 40‐year‐old female with AMA and ANA positivity without fulfilling definitive AIH/PBC criteria. While a gluten‐free diet often resolves liver abnormalities, some patients may develop MASLD due to high‐glycemic substitutes or may require immunosuppressants for concurrent autoimmune liver disease. This highlights the need for liver screening in patients with CD, even when asymptomatic, to ensure early diagnosis and tailored management. Further research is warranted to clarify the mechanisms linking gluten sensitivity, autoimmunity, and liver injury to optimize therapeutic approaches for these complex presentations.

## Author Contributions

**Sana Muhammad Hussain:** conceptualization, supervision, writing – original draft. **Madiha Khan:** data curation, investigation, methodology, resources, writing – original draft, writing – review and editing. **Zahra Anas:** conceptualization, methodology, supervision, writing – original draft, writing – review and editing. **Zarlish Khan:** investigation, supervision, writing – original draft, writing – review and editing. **Amanullah Abbasi:** conceptualization, project administration, resources, writing – original draft, writing – review and editing. **Md Ariful Haque:** formal analysis, visualization, writing – review and editing.

## Funding

The authors have nothing to report.

## Ethics Statement

The authors have nothing to report.

## Consent

Written informed consent was obtained from the patient for publication and accompanying images. A copy of the written consent is available for review by the editor‐in‐chief of this journal on request.

## Conflicts of Interest

The authors declare no conflicts of interest.

## Data Availability

The authors have nothing to report.
